# Effect of Concentrated Salts Solutions on the Stability of Immobilized Enzymes: Influence of Inactivation Conditions and Immobilization Protocol

**DOI:** 10.3390/molecules26040968

**Published:** 2021-02-12

**Authors:** Sabrina Ait Braham, El-Hocine Siar, Sara Arana-Peña, Diego Carballares, Roberto Morellon-Sterling, Hossein Bavandi, Diandra de Andrades, Jakub F. Kornecki, Roberto Fernandez-Lafuente

**Affiliations:** 1 Departamento de Biocatálisis, ICP-CSIC, Campus UAM-CSIC, 28049 Madrid, Spain; sabrina.aitbraham@yahoo.fr (S.A.B.); hocines1@hotmail.fr (E.-H.S.); s.arana@csic.es (S.A.-P.); diego.carballares@csic.es (D.C.); r.m.sterling@csic.es (R.M.-S.); hosein.bavandi@yahoo.com (H.B.); dcandrades@gmail.com (D.d.A.); 2 Laboratoire de Biotechnologies Végétales et Ethnobotanique, Faculté des Sciences de la Nature et de la Vie, Université de Bejaia, Bejaia 06000, Algeria; 3 Transformation and Food Product Elaboration Laboratory, Nutrition and Food Technology Institute (INATAA), University of Brothers Mentouri Constantine 1, Constantine 25017, Algeria; 4 Department of Pure Chemistry, Faculty of Chemistry, Shahid Beheshti University, G.C., Tehran 1983969411, Iran; 5 Biotechnology, Bioprocess, and Biocatalysis Group, Food Science and Technology Institute, Federal University of Rio Grande do Sul, Porto Alegre 90040-060, RS, Brazil

**Keywords:** immobilized enzyme, tuning enzyme properties by immobilization, enzyme stability, enzyme stabilization, ionic strength

## Abstract

This paper aims to investigate the effects of some salts (NaCl, (NH_4_)_2_SO_4_ and Na_2_SO_4_) at pH 5.0, 7.0 and 9.0 on the stability of 13 different immobilized enzymes: five lipases, three proteases, two glycosidases, and one laccase, penicillin G acylase and catalase. The enzymes were immobilized to prevent their aggregation. Lipases were immobilized via interfacial activation on octyl agarose or on glutaraldehyde-amino agarose beads, proteases on glyoxyl agarose or glutaraldehyde-amino agarose beads. The use of high concentrations of salts usually has some effects on enzyme stability, but the intensity and nature of these effects depends on the inactivation pH, nature and concentration of the salt, enzyme and immobilization protocol. The same salt can be a stabilizing or a destabilizing agent for a specific enzyme depending on its concentration, inactivation pH and immobilization protocol. Using lipases, (NH_4_)_2_SO_4_ generally permits the highest stabilities (although this is not a universal rule), but using the other enzymes this salt is in many instances a destabilizing agent. At pH 9.0, it is more likely to find a salt destabilizing effect than at pH 7.0. Results confirm the difficulty of foreseeing the effect of high concentrations of salts in a specific immobilized enzyme.

## 1. Introduction

Enzymes are the most effective, selective and specific catalysts in Nature [1,2,3,4,5]. These features make them the best options for the requirements of green chemistry, as they can catalyze a complex process under the mildest experimental conditions [6]. However, they have evolved to fulfill some physiological requirements (e.g., to give a fast answer under stress situations) and some of their features do not fit those of an industrial biocatalyst: maintain high activity, stability, selectivity and specificity for long periods of time under conditions quite far from the physiological ones and on synthetic substrates. These enzyme limitations may be avoided in some instances by the great advances that have been achieved in the last decades in some disciplines related to biocatalyst design, such as metagenomics [7,8,9,10], enzyme modelling and site-directed mutagenesis [11,12], directed evolution [13,14,15,16], enzyme chemical or physical modification [17,18,19], enzyme immobilization [20,21,22] or reactor design [23,24,25,26]. Some instances show the combination of several of these techniques to get synergetic effects [27,28,29]. For example, in a very nice example of the use of several techniques, an esterase was supplemented with an additional artificial active center (creating the so-called plurizymes) via enzyme modelling and side-directed mutagenesis [30], its activity was later improved by the same tools [31], and an irreversible inhibitor bearing a catalytic organo-metal complex was designed for one of the active centers and coupled to it, enabling the use of just one enzyme molecule to catalyze a cascade process with an enzyme and a metallic active centers in the same protein molecule [31].

Medium design also plays an important role in determining enzyme stability [32,33,34,35,36]. For example, enzymes may be submitted to (or even be used in) media bearing high concentrations of salts. This is a situation that occurs sometimes during the handling of enzymes. For example, when the enzymes are purified via ion exchange, the most strongly adsorbed proteins may require a high concentration of salts to become desorbed from the column [37,38,39]. Another example where the enzymes may be exposed to high salt concentrations is the fractioning of protein extracts by selective precipitation using ammonium sulfate solutions at different saturation percentages [40,41]. Similarly, enzymes may be purified by using salt/polymer aqueous biphasic systems [42,43,44,45], or in some instances these aqueous biphasic systems may become the reaction medium where the enzyme is finally used [46,47,48,49,50,51,52,53,54,55]. In all these examples, the enzymes will be exposed to high ion strength if used in (or extracted to) the salt phase. 

While the effects of many additives on enzyme stability have been studied in more or less detail [56], the effect of high ion strength on enzyme stability has not been hitherto studied systematically. One reason for this lack of studies is that changes in the ionic strength may force enzyme aggregations when using soluble enzymes, and that can make understanding of the actual effects of these concentrated salts solutions on enzyme stability complex [57,58]. Some of these studies are just theoretical [59,60] and have reached different conclusions. For example, in one paper the researchers stated that an increased ionic strength may reduce the intensity of the protein ion bridges. In some instances, the ion bridges stabilize the partially inactivated form of the protein with a higher intensity than the native one, and this reduces the enzyme stability. In these specific cases, the weakening of the ion bridges may be positive for the enzyme stability [61]. In another example, using the three-dimensional structure information and some modelling of the proteins, the negative effect of increasing the ionic strength at acid pH on the stability of sperm whale apomyoglobin was related to the decrease of attractive charge-charge interactions which destabilize more the native state of the enzyme than a compact enzyme intermediate formed during its inactivation [62]. In another paper, three proteins obtained from mesophilic, thermophilic and hyperthermophilic bacteria were employed to study the effect of salt concentration on protein stability using continuum electrostatic models [63]. The model shows that the mesophilic protein should be stabilized in the presence of high salt concentration while the thermophile and hyperthermophile enzymes should be destabilized. Other papers analyzed the solubility of different amino acids and some model compounds at increasing ionic strength, trying to correlate this with the effect of the ion strength on enzyme stability [64,65]. In an experimental paper, the dimeric alkaline phosphatase from *Vibrio splendidus* was found to be extremely unstable at low ionic strengths, and the enzyme stability increased when the concentration of NaCl was increased, although the ionic strength effect on the enzyme stability was pH dependent [66]. That way, the effect of the ionic strength on enzyme stability is quite complex and unclear to date [64], with very few experimental research in the matter.

The complexity of this subject increases considering that in certain cases specific cations/anions are relevant for enzyme stability. Some enzymes stabilities, like the multimeric *β*-galactosidases from *Escherichia coli* or *Kluyveromyces lactis* [67,68,69], depend on some cations that are critical to maintain the assembly of the subunits, and these cations may be released in the presence of high concentrations of other cations. Similarly, phosphate anions are critical for the stability of the multimer stability of the amino acid ester hydrolase from *Acetobacter turbidans* [70]. Zn^2+^ was found to be critical for the stability of the multimeric catalase from *Aspergillus niger*, and this effect was not related to the stability of the multimer, as this effect was found even after the prevention of enzyme subunits dissociation via multisubunit immobilization and further crosslinking [71].

The nature of the salt is also important. For example, lipase from *Thermomyces lanuginosus* immobilized on octyl agarose is stabilized by high concentrations of NaCl [72] but it is destabilized by sodium phosphate [73]. The fact that, in some instances, the effect of the nature of the buffer on the enzyme stability depends on the way the enzyme is immobilized as well as on the inactivation pH, makes the understanding of this effect more complex. In fact, some cations stabilize lipases immobilized on octyl agarose, but not when they are immobilized on other supports [74,75]. Similarly, the effects of moderate concentrations of phosphate anions on the stability of lipases immobilized on octyl agarose are always negative when inactivated at pH 7.0, but this effect is not general for all lipases when using other immobilization strategies or inactivation pH values [76]. Moreover, the presence of high concentrations of NaCl reduced these negative effects of the phosphate anions [76]. This was explained because while the lipases that have been immobilized on octyl agarose presented the lipase in its stabilized open form [77,78], the covalently immobilized enzyme maintains the open/closed conformational equilibrium [79,80,81].

This new paper tries to compare the effect of the high ionic strength on enzyme stabilities, employing different salts, but using immobilized enzymes to avoid enzyme aggregation that can make understanding the results difficult. The stress inactivations have been performed at different pH values and using different cations and anions to analyze the effect of the nature of the salt (NaCl, (NH_4_)_2_SO_4_ and Na_2_SO_4_). In the case of lipases, lipases immobilized on octyl agarose or glutaraldehyde-amino agarose have been employed, as this seems to alter the lipase features response to changes in the medium as stated above [56,57,58,64,65,67,68,69,70,72,73,74,76]. As lipases, this study includes the lipases A and B from *Candida antarctica* (CALA and CALB) [82,83,84,85,86,87,88], and the lipases from *Candida rugosa* (CRL) [89] and from *Rhizomucor miehei* (RML) [90,91]. Moreover, Eversa^®^ Transform 2.0 (EVT), a recombinant commercial enzyme that has been improved from the initial lipase from *Thermomyces lanuginosus* to improve their performance in biodiesel production [92,93] has been also studied. The proteases ficin from *Ficus carica* [94,95], chymotrypsin from bovine liver [96,97] and trypsin from bovine liver [98,99] have been immobilized on glyoxyl agarose [100,101] and glutaraldehyde-amino agarose beads [102]. The study also includes other monomeric enzymes and some multimeric enzymes, that have been immobilized on glutaraldehyde-amino agarose [102], such as *β*-galactosidase from *Aspergillus oryzae* [103] and laccase from *Myceliophthora thermophila* [104], a dimeric enzyme such as *β*-glucosidase from *Aspergillus niger* [105,106] and the tetrameric catalase from bovine liver [107]. Finally, the penicillin G acylase from *Escherichia coli* (PGA) [108,109,110] immobilized in glyoxyl agarose [100,101] has been included in this study.

## 2. Results and Discussion

### 2.1. Effect of Different Salts on the Stability of Immobilized Lipases

As stated in the introduction section, lipases were immobilized on octyl agarose (via interfacial activation [77]) or glutaraldehyde-amino agarose (via multiple factors) [111], to have two immobilized preparations via quite different phenomena. 

**Figure 1:**
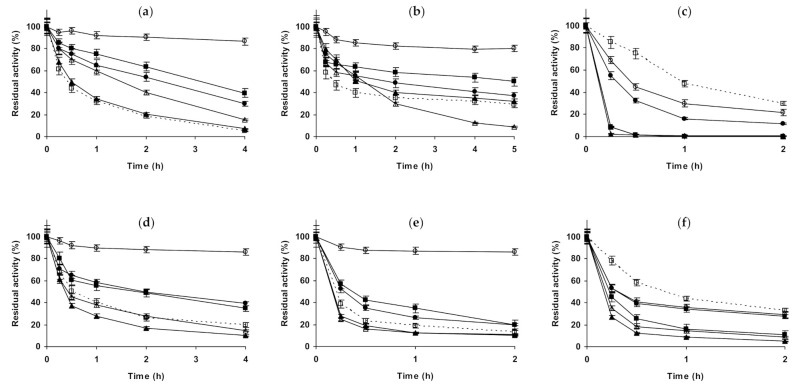
Effect of ionic strength and inactivation pH on the stability of different immobilized preparations of CALA. Octyl-CALA: (**a**) pH 5.0 and 87 °C; (**b**) pH 7.0 and 82 °C; (**c**) pH 9.0 and 55 °C. Glutaraldehyde-CALA: (**d**) pH 5.0 and 83 °C; (**e**) pH 7.0 and 87 °C; (**f**) pH 9.0 and 43 °C. Other specifications are described in the Methods section. Dotted line, empty squares: references; full triangles: 1 M NaCl; empty triangles: 3 M NaCl; full circles: 1 M (NH_4_)_2_SO_4_; empty circles: 3 M (NH_4_)_2_SO_4_ and full squares: 1 M Na_2_SO_4_.

Starting with CALA (Figure 1), when the enzyme is immobilized on octyl agarose (Figure 1a–c), and with its inactivation at pH 5.0, the presence of 1 M NaCl presented no effect on enzyme stability, while using 3 M of this salt, the stability was slightly improved. (NH_4_)_2_SO_4_ presented a very positive effect on enzyme stability, which reached a maximum using 3 M. In the presence of 1 M Na_2_SO_4_, the biocatalyst presented a similar stability to that in the presence of 1 M (NH_4_)_2_SO_4_. At pH 7.0, 1 M NaCl had no significant effect on enzyme stability, while in the presence of 3 M NaCl there was an initial stabilizing effect, but after 5 h the residual activity was under that of the enzyme incubated in absence of additional salts. (NH_4_)_2_SO_4_ remained a stabilizing medium for this immobilized enzyme at pH 7.0, being this effect more significant at 3 M, 1 M Na_2_SO_4_ gave similar value that 1 M (NH_4_)_2_SO_4_. At pH 9.0, all concentrated salts strongly reduced the stability of octyl-CALA. The most drastic destabilizing effect was found using the sodium salts (sulfate or chloride), (NH_4_)_2_SO_4_ showed a lower destabilizing effect. Curiously, considering the negative effect of the salt, 3 M concentration gave higher stability than 1 M of the salts (Figure 1c). Using glutaraldehyde-CALA (Figure 1d–f), at pH 5.0, NaCl showed a slight destabilizing effect, more relevant in the presence of 1 M NaCl than using 3 M. The enzyme under these conditions was significantly stabilized by (NH_4_)_2_SO_4_, even more so using 3 M than employing 1 M. Both 1 M sulfate salts permitted the same stabilities for this immobilized enzyme. At pH 7.0, NaCl had a slightly negative effect on enzyme stability at 1 and 3 M. 1 M Na_2_SO_4_ showed a slight stabilizing effect, similar to 1 M (NH_4_)_2_SO_4_. However, the inactivation in 3 M (NH_4_)_2_SO_4_ gave the highest stability, even more relevant than using octyl-CALA (Figure 1b). At pH 9.0, as in the case of octyl-CALA, a negative effect on enzyme stability of all concentrated salts was observed, being this effect more significant for both sodium salts than for (NH_4_)_2_SO_4_.

**Figure 2:**
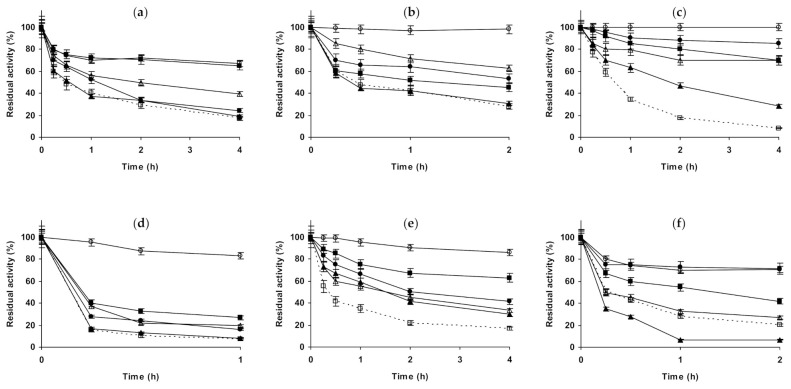
Effect of ionic strength and inactivation pH on the stability of different immobilized preparations of CALB. Octyl-CALB: (**a**) pH 5.0 and 83 °C; (**b**) pH 7.0 and 80 °C; (**c**) pH 9.0 and 58 °C. Glutaraldehyde-CALB: (**d**) pH 5.0 and 78 °C; (**e**) pH 7.0 and 70 °C; (**f**) pH 9.0 and 60 °C. Other specifications are described in the Methods section. Dotted line, empty squares: references; full triangles: 1 M NaCl; empty triangles: 3 M NaCl; full circles: 1 M (NH_4_)_2_SO_4_; empty circles: 3 M (NH_4_)_2_SO_4_ and full squares: 1 M Na_2_SO_4_.

Next, we will present the results using immobilized CALB (Figure 2). In the case of octyl-CALB (Figure 2a–c) at pH 5.0, 3M NaCl produced a positive effect on enzyme stability, while 1 M had no significant effect. Using (NH_4_)_2_SO_4_, some enzyme stabilization could be detected using 1 M, while 3 M of this salt was much more stabilizing, more than 3 M NaCl. 1 M Na_2_SO_4_ presented a significant effect on enzyme stability, similar to the value found using 3 M (NH_4_)_2_SO_4_. At pH 7.0, 1 M NaCl had no effects on enzyme stability, while 3 M significantly improved it. (NH_4_)_2_SO_4_ was positive for enzyme stability using 1 M, but when using 3 M the effect was much more significant, the enzyme almost remained fully active after 2 h of incubation. At this pH value, 1 M Na_2_SO_4_ had a lower stabilizing effect than 1 M (NH_4_)_2_SO_4_. At pH 9.0, results fully differed from those described using immobilized CALA: the stability increased using all additives. NaCl and (NH_4_)_2_SO_4_ increased the stability of the enzyme when increasing their concentration. The most stabilizing agent was (NH_4_)_2_SO_4_ followed by Na_2_SO_4_ and the least stabilizing agent was NaCl (comparing to the effects of the other salts at 1 M). Using glutaraldehyde-CALB (Figure 2d–f), at pH 5.0, 1 M NaCl had no effect on enzyme stability and for 3 M NaCl a slight stabilizing affect was found. 1 M Na_2_SO_4_ presented a higher stabilization effect than 3 M NaCl, while 1 M (NH_4_)_2_SO_4_ had scarce stabilizing effect, very similar at that found using 3 M NaCl. The clearest stabilizing effect could be found when inactivating the immobilized enzyme in 3 M (NH_4_)_2_SO_4_. This was quite different from the results found using octyl-CALB (Figure 2a). At pH 7.0, all salts at all concentrations improved enzyme stability. The presence of 1 and 3 M NaCl presented a similar effect on enzyme stability, much smaller than when using the sulfate salts. 1 M (NH_4_)_2_SO_4_ stabilized this biocatalyst to a lower extent the enzyme than 1 M Na_2_SO_4_, however the highest stability was observed using 3 M (NH_4_)_2_SO_4_. Again, there are some qualitative differences compared to the effects of the salts in the stability of the octyl-CALB preparation (Figure 2b). At pH 9.0, glutaraldehyde-CALB stability decreased when inactivated in the presence of 1 M of NaCl, becoming the immobilized enzyme stability similar when inactivated in absence of any salt or in the presence of 3 M NaCl. 1 M Na_2_SO_4_ presented some stabilizing effects, although smaller than 1 M (NH_4_)_2_SO_4_. This salt gave the same stability when used at 1 or 3 M. Again, this was different to the results using octyl-CALB (Figure 2c).

**Figure 3:**
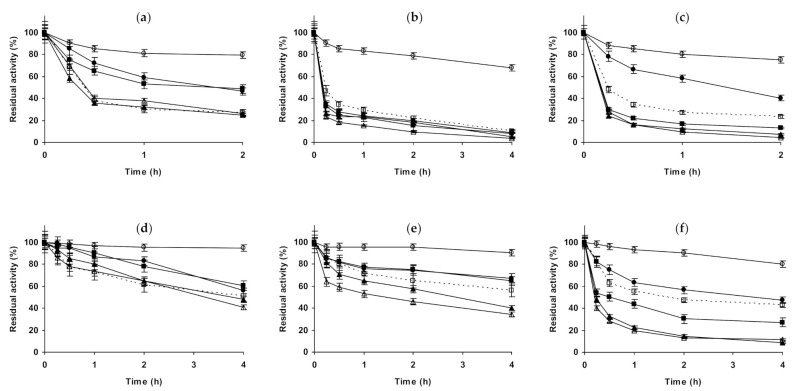
Effect of ionic strength and inactivation pH on the stability of different immobilized preparations of CRL. Octyl-CRL: (**a**) pH 5.0 and 65 °C; (**b**) pH 7.0 and 64 °C; (**c**) pH 9.0 and 40 °C. Glutaraldehyde-CRL: (**d**) pH 5.0 and 68 °C; (**e**) pH 7.0 and 67 °C; (**f**) pH 9.0 and 40 °C. Other specifications are described in the Methods section. Dotted line, empty squares: references; full triangles: 1 M NaCl; empty triangles: 3 M NaCl; full circles: 1 M (NH_4_)_2_SO_4_; empty circles: 3 M (NH_4_)_2_SO_4_ and full squares: 1 M Na_2_SO_4_.

Next, we studied CRL (Figure 3). In the case of octyl-CRL (Figure 3a–c), at pH 5.0, the stability did not change in the presence of 1 or 3 M NaCl. Sulfate salts presented a positive effect, similar using 1 M of ammonium or sodium salts, more significant using 3 M (NH_4_)_2_SO_4_. At pH 7.0, NaCl presented a slight negative effect on the immobilized octyl-CRL stability. This effect was stronger using 3 M. 1 M Na_2_SO_4_ or (NH_4_)_2_SO_4_ were also slightly negative for the enzyme stability. However, 3 M (NH_4_)_2_SO_4_ clearly stabilized the enzyme. At pH 9.0, the negative effect of NaCl remained (similar at both, 1 or 3 M), 1 M Na_2_SO_4_ was also negative for enzyme stability. However, (NH_4_)_2_SO_4_ presented positive effects on enzyme stability, and these positive effects increased with the salt concentration. The effect of these salts in the glutaraldehyde-CRL stability was also analyzed (Figure 3d–f). At pH 5.0, NaCl had no significant effect on the stability of the enzyme (similar to the octyl preparation). 1 M Na_2_SO_4_ or (NH_4_)_2_SO_4_ increased the enzyme stability in a similar way, but the highest stabilization was found using 3 M (NH_4_)_2_SO_4_. At pH 7.0, this biocatalyst was slightly destabilized by NaCl, and this effect was higher when the salt concentration increased. 1 M Na_2_SO_4_ or (NH_4_)_2_SO_4_ presented a marginal stabilizing effect, this stabilizing effect became more evident using 3 M (NH_4_)_2_SO_4_. When the immobilized enzyme was inactivated at pH 9.0, the enzyme destabilizing effect of NaCl was more intense than at the other pH values. 1 M Na_2_SO_4_ was slightly destabilizing while the immobilized enzyme in the presence of 1 M (NH_4_)_2_SO_4_ was more stable than in just buffer. This (NH_4_)_2_SO_4_ stabilizing effect was further increased using 3 M of the salt. Results were similar, but not identical, to the ones obtained studying the octyl-CRL preparation (Figure 3a–c), but very different from the results observed using the other two lipases already discussed (Figure 1 and Figure 2).

**Figure 4:**
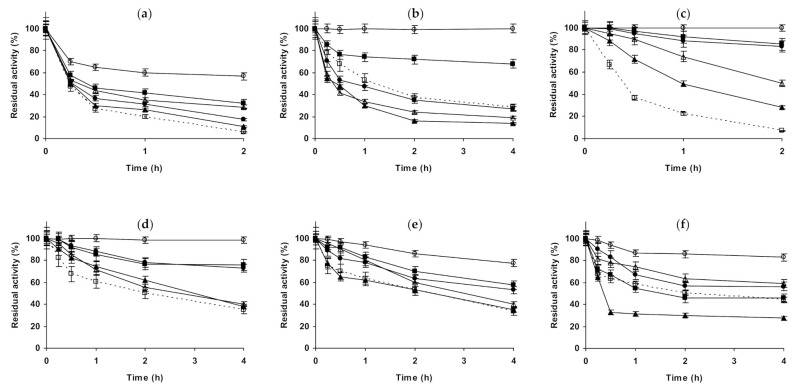
Effect of ionic strength and inactivation pH on the stability of different immobilized preparations of RML. Octyl-RML: (**a**) pH 5.0 and 62 °C; (**b**) pH 7.0 and 60 °C; (**c**) pH 9.0 and 45 °C. Glutaraldehyde-RML: (**d**) pH 5.0 and 64 °C; (**e**) pH 7.0 and 62 °C; (**f**) pH 9.0 and 63 °C. Other specifications are described in the Methods section. Dotted line, empty squares: references; full triangles: 1 M NaCl; empty triangles: 3 M NaCl; full circles: 1 M (NH_4_)_2_SO_4_; empty circles: 3 M (NH_4_)_2_SO_4_ and full squares: 1 M Na_2_SO_4_.

The next studied lipase was RML (Figure 4). Using octyl-RML (Figure 4a–c), at pH 5.0, the presence of 1 or 3 M NaCl had some stabilizing effect on this enzyme; clearer at 3 M. 1 M Na_2_SO_4_ was more stabilizing than 1 M (NH_4_)_2_SO_4_ or 3 M NaCl, the highest enzyme stability was observed using 3 M (NH_4_)_2_SO_4_. At pH 7.0, NaCl presented a negative effect on enzyme stability, being the inactivating results quite similar to the biocatalyst in 1 or 3 M of this salt. 1 M Na_2_SO_4_ effect was stabilizing, while 1 M (NH_4_)_2_SO_4_ was slightly destabilizing for this enzyme preparation. However, when the inactivation was performed in 3 M (NH_4_)_2_SO_4_, the enzyme stability reached a maximum (no loss of activity was detected under these conditions for the time of study). At pH 9.0, NaCl produced a stabilization of the enzyme, more significant when the concentration of the salt was increased. 1 M Na_2_SO_4_ or (NH_4_)_2_SO_4_ stabilized the enzyme more than 3 M NaCl, and in a very similar fashion. The highest stability was observed in 3 M (NH_4_)_2_SO_4_. Using glutaraldehyde-RML (Figure 4d–f), at pH 5.0, NaCl presented a small stabilizing effect using both concentrations, 1 and 3 M. Using 1 M of both sulfate salts, the stabilizing effect was clearer and almost identical. Again, the highest stabilizing effect was obtained using 3 M (NH_4_)_2_SO_4_. At pH 7.0, the presence of 1 M NaCl had no significant effect on the enzyme stability, while 3 M produced a slight stabilization. The stability of the enzyme further improved in the presence of 1 M Na_2_SO_4_, and to a lower extent in the presence of 1 M (NH_4_)_2_SO_4_. The biocatalyst inactivation in 3 M (NH_4_)_2_SO_4_ produced the lower inactivation rate. At pH 9.0, 1 M NaCl produced a negative effect on enzyme stability, while 3 M NaCl produced a positive effect. 1 M Na_2_SO_4_ presented no effect on enzyme stability, while 1 M (NH_4_)_2_SO_4_ presented a stabilizing effect, that was augmented using 3 M of this salt. Again, results were diverse depending on the pH and on the enzyme immobilization protocol.

**Figure 5:**
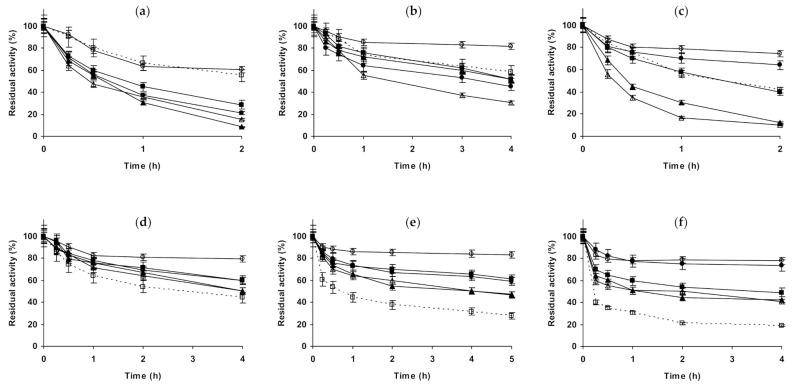
Effect of ionic strength and inactivation pH on the stability of different immobilized preparations of EVT. Octyl-EVT: (**a**) pH 5.0 and 77 °C; (**b**) pH 7.0 and 75 °C; (**c**) pH 9.0 and 73 °C. Glutaraldehyde-EVT: (**d**) pH 5.0 and 75 °C; (**e**) pH 7.0 and 76 °C; (**f**) pH 9.0 and 68 °C. Other specifications are described in the Methods section. Dotted line, empty squares: references; full triangles: 1 M NaCl; empty triangles: 3 M NaCl; full circles: 1 M (NH_4_)_2_SO_4_; empty circles: 3 M (NH_4_)_2_SO_4_ and full squares: 1 M Na_2_SO_4_.

The last lipase that has been studied in this paper was EVT (Figure 5). Using octyl-EVT (Figure 5a–c), at pH 5, 1 and 3 M NaCl presented a similar negative effect on enzyme stability. In the presence of 1 M Na_2_SO_4_, the enzyme stability was reduced in a smaller way, results were slightly worse using 1 M (NH_4_)_2_SO_4_, while using 3 M of this salt the enzyme stability was similar to the inactivation performed just in buffer. At pH 7.0, the inactivation in the presence of 1 M NaCl or Na_2_SO_4_ presented no differences on enzyme stability, while 3 M NaCl was clearly negative for enzyme stability. 1 M (NH_4_)_2_SO_4_ was slightly negative for enzyme stability, while 3 M of this salt presented a stabilizing effect. At pH 9.0, NaCl presented a negative effect on enzyme stability, 1 M Na_2_SO_4_ has no significant effect on enzyme stability, and (NH_4_)_2_SO_4_ presented an enzyme stabilizing effect, higher using 3 M than employing 1 M of this salt. Figure 5d–f show the results using glutaraldehyde-EVT. At pH 5.0, NaCl presented a small stabilizing effect, similar using 1 or 3 M. Using 1 M Na_2_SO_4_ or (NH_4_)_2_SO_4_, the stabilization of the enzyme was more evident, and this stabilizing effect increased using 3 M of (NH_4_)_2_SO_4_, although it did not become very relevant (e.g., after 4 h, the reference maintained 44% of the initial activity while in the presence of 3 M (NH_4_)_2_SO_4_ maintained almost 80%). At pH 7.0, the enzyme stabilizing effect of NaCl is more evident, again very similar at 1 or 3 M. 1 M Na_2_SO_4_ or (NH_4_)_2_SO_4_ further improved the enzyme stability, and the highest stability was observed in inactivations using 3 M (NH_4_)_2_SO_4_. At pH 9.0, the presence of 1 or 3 M NaCl improved the enzyme stability in a similar fashion. 1 M Na_2_SO_4_ permitted stabilities similar to that observed when inactivating the immobilized enzyme in the presence of NaCl, while the inactivation in (NH_4_)_2_SO_4_ gave the highest enzyme stabilities, very similar using 1 or 3 M of these salts.

Thus, the results presented here suggest that the complexity of the effects of the salts in lipase stability is very diverse and a single explanation cannot justify the great differences among enzymes, inactivation pH and immobilization protocol. The different effect of the different salts on the immobilized lipases stability should be due to the different structure of the enzymes, in a similar way that Ca^2+^ stabilized some lipases and not others, the different cations and anions may promote different effects on the enzyme stability. As a general, but not universal rule (see in Figure 1 the case of CALA at pH 9.0), 3 M (NH_4_)_2_SO_4_ generally stabilize lipases, while NaCl is more risky. In fact, NaCl is even negative for the stability of some of the analyzed immobilized enzymes under certain conditions. The explanation is not always because of the Na^+^ cations, as in some instances NaCl is destabilizing while Na_2_SO_4_ is stabilizing, even more than (NH_4_)_2_SO_4_. The fact that in some instances the negative effect of the salt decreases when increasing its concentration, even becoming positive in certain cases, suggests that a double effect of the salts on the enzyme stability may be occurring in these cases, one negative, perhaps related to the effect of the specific ions, and another positive, perhaps related to an increase in the ionic strength that makes the exposition of partially distorted enzymes, where some internal hydrophobic pockets may be exposed to the medium, less favorable [57,58,59,60,61,62,63,64]. Differences between the different immobilized preparations of the same enzyme may derive from the fact that when immobilized in hydrophobic support, the lipases present the open form of the lipase, that is stabilized *versus* the hydrophobic support surface, while in the glutaraldehyde-amino, this did not occur [78,102]. Moreover, in the enzymes immobilized via interfacial activation, the enzyme release from the biocatalyst at high temperature should be more difficult at growing ionic strength, and this effect will not occur using covalently immobilized lipases [78].

### 2.2. Effect of Different Salts on the Stability of Immobilized Proteases

To determine if this quite apparently chaotic effect of the presence of salts on enzyme stabilities is general or specific for lipases, we have also analyzed the stability of 3 proteases immobilized on glutaraldehyde-amino [102] and on glyoxyl agarose [101], protocols that should give different enzyme orientations and degrees of enzyme-support multipoint covalent attachment.

**Figure 6:**
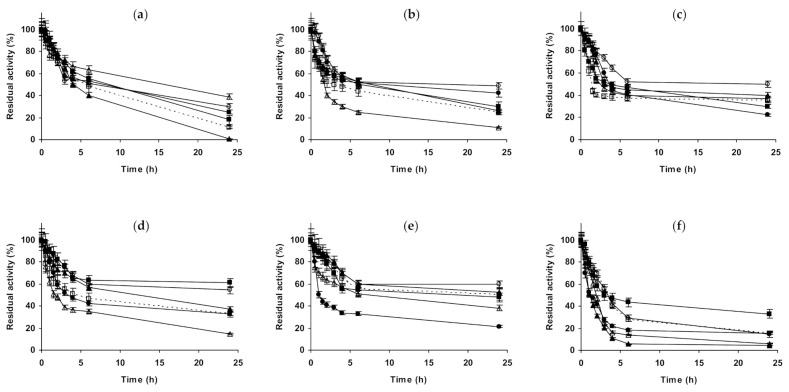
Effect of ionic strength and inactivation pH on the stability of different immobilized preparations of ficin. Glyoxyl-ficin: (**a**) pH 5.0 and 60 °C; (**b**) pH 7.0 and 60 °C; (**c**) pH 9.0 and 60 °C. Glutaraldehyde-ficin: (**d**) pH 5.0 and 60 °C; (**e**) pH 7.0 and 60 °C; (**f**) pH 9.0 and 60 °C. Other specifications are described in the Methods section. Dotted line, empty squares: references; full triangles: 1 M NaCl; empty triangles: 3 M NaCl; full circles: 1 M (NH_4_)_2_SO_4_; empty circles: 3 M (NH_4_)_2_SO_4_ and full squares: 1 M Na_2_SO_4_.

Starting with glyoxyl-ficin (Figure 6a–c), the presence of 1 M NaCl at pH 5.0 presented a small enzyme destabilizing effect, while 3 M NaCl was significantly stabilizing for this preparation (the enzyme inactivated under these conditions was the most stable). 1 M Na_2_SO_4_ or (NH_4_)_2_SO_4_ presented a similar and small stabilizing effect, similar to that found using 3 M (NH_4_)_2_SO_4_. At pH 7.0, 1 M NaCl, sodium and (NH_4_)_2_SO_4_, or 3 M (NH_4_)_2_SO_4_ presented very small stabilizing effects, while 3 M NaCl was clearly negative for the enzyme stability (in opposition with the results at pH 5). At pH 9.0, the presence of the salts produced a small stabilizing effect, reaching the highest one when inactivating in 3 M (NH_4_)_2_SO_4_. 1 M (NH_4_)_2_SO_4_ and Na_2_SO_4_ were slightly destabilizing after 24 h of inactivation. When using glutaraldehyde-ficin (Figure 6d–f), the differences in the stabilities of the biocatalysts in the different solutions were larger than when using glyoxyl-ficin (Figure 6a–c), although not very great. At pH 5.0, 1 M NaCl was slightly stabilizing, while 3 M NaCl was destabilizing for this ficin preparation (this was the contrary using glyoxyl-ficin, Figure 6a). The medium stabilizing effect for this biocatalyst was increased using 1 M Na_2_SO_4_, while 1 M (NH_4_)_2_SO_4_ presented no-effect on enzyme stability and 3 M (NH_4_)_2_SO_4_ gave a stabilization similar to that using 1 M Na_2_SO_4_. At pH 7.0, the effects of NaCl were similar to those found at pH 5.0, 1 M slightly stabilize the enzyme, while 3 M slightly destabilized the enzyme. 1 M Na_2_SO_4_ had no effect on enzyme stability, while 1 M (NH_4_)_2_SO_4_ was clearly negative and 3 M of this salt had a very small positive effect on enzyme stability. At pH 9.0, NaCl was negative for enzyme stability at both, 1 and 3 M. 1 M Na_2_SO_4_ was positive for enzyme stability while 1 M (NH_4_)_2_SO_4_ was negative, and using 3 M (NH_4_)_2_SO_4_ the enzyme stability was very similar to that found just in buffer.

**Figure 7:**
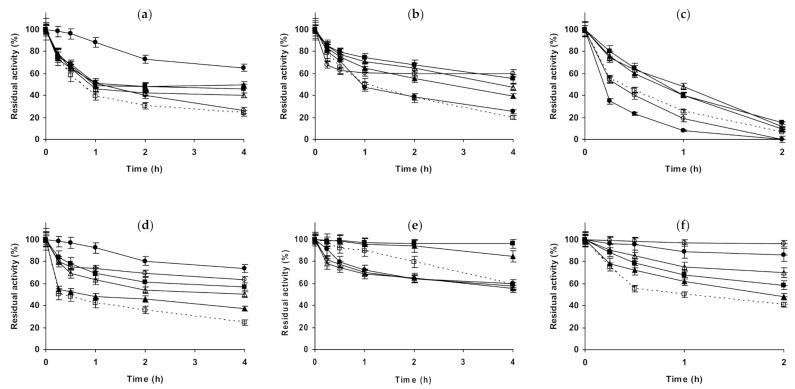
Effect of ionic strength and inactivation pH on the stability of different immobilized preparations of chymotrypsin. Glyoxyl-chymotrypsin: (**a**) pH 5.0 and 83 °C; (**b**) pH 7.0 and 77 °C; (**c**) pH 9.0 and 70 °C. Glutaraldehyde-chymotrypsin: (**d**) pH 5.0 and 56 °C; (**e**) pH 7.0 and 54 °C; (**f**) pH 9.0 and 44 °C. Other specifications are described in the Methods section. Dotted line, empty squares: references; full triangles: 1 M NaCl; empty triangles: 3 M NaCl; full circles: 1 M (NH_4_)_2_SO_4_; empty circles: 3 M (NH_4_)_2_SO_4_ and full squares: 1 M Na_2_SO_4_.

Figure 7 shows the studies using immobilized chymotrypsin. Glyoxyl-chymotrypsin (Figure 7a–c), at pH 5.0 and in the presence of 1 M NaCl and 1 M Na_2_SO_,_ presented a similar increase of enzyme stability. If the inactivation was performed in 3 M NaCl, the stabilizing effect was smaller. 1 M (NH_4_)_2_SO_4_ permitted much higher enzyme stabilization than the other salts, but this effect became similar to the other salts when using 3 M (NH_4_)_2_SO_4_. At pH 7.0, NaCl presented a positive effect that was higher using 3 M NaCl. 1 M Na_2_SO_4_ produced a higher stabilization effect, while 1 M (NH_4_)_2_SO_4_ did not produce a significant effect on enzyme stability. Using 3 M (NH_4_)_2_SO_4_, after a first rapid inactivation, the activity of the enzyme did not decrease for a long time, after 4 h the enzyme residual activity became even higher than in 1 M Na_2_SO_4_. At pH 9.0, again NaCl produced a positive effect on enzyme stability, slightly higher using 3 M. 1 M Na_2_SO_4_ is positive for enzyme stability, while 1 M (NH_4_)_2_SO_4_ reduced the enzyme stability, decreasing this negative effect on enzyme stability when using 3 M of this salt. Using glutaraldehyde-chymotrypsin (Figure 7d–f), the situation is very different. At pH 5.0, NaCl produced enzyme stabilization, higher using 3 M. 1 M Na_2_SO_4_ produced an even higher stabilization than when using 3 M NaCl. The highest enzyme stabilization is achieved using 1 M (NH_4_)_2_SO_4_, the use of 3 M of this salt is positive for immobilized enzyme stability, but in a smaller extension. At pH 7.0, 1 M NaCl produced a very positive effect on enzyme stability, while 3 M NaCl produced the enzyme destabilization. 1 M Na_2_SO_4_ promoted a strong enzyme stabilization, slightly higher than when using 1 M NaCl (residual activity was 95 versus 85% after 4 h of inactivation). (NH_4_)_2_SO_4_ was negative for enzyme stability, at both concentrations with similar intensity, and similar to the results obtained using 3 M NaCl. At pH 9.0, NaCl produced a positive effect on enzyme stability, higher using 3 M. 1 M Na_2_SO_4_ produced an enzyme stabilizing effect slightly smaller than 3 M NaCl, while (NH_4_)_2_SO_4_ promoted the highest enzyme stabilization, higher when using higher salt concentration.

**Figure 8:**
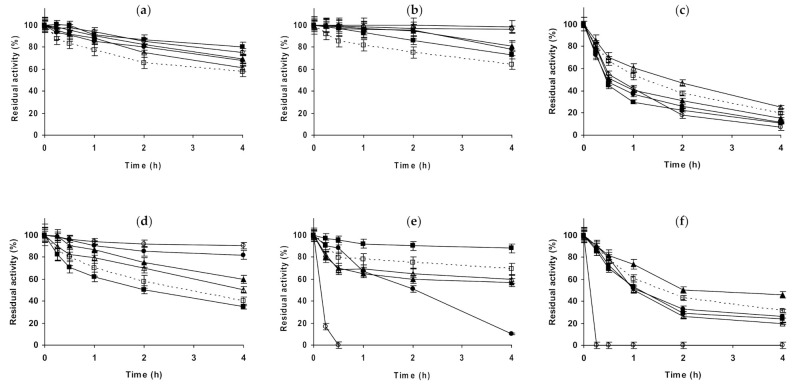
Effect of ionic strength and inactivation pH on the stability of different immobilized preparations of trypsin. Glyoxyl-trypsin: (**a**) pH 5.0 and 82 °C; (**b**) pH 7.0 and 79 °C; (**c**) pH 9.0 and 70 °C. Glutaraldehyde-trypsin: (**d**) pH 5.0 and 52 °C; (**e**) pH 7.0 and 51 °C; (**f**) pH 9.0 and 42 °C. Other specifications are described in the Methods section. Dotted line, empty squares: references; full triangles: 1 M NaCl; empty triangles: 3 M NaCl; full circles: 1 M (NH_4_)_2_SO_4_; empty circles: 3 M (NH_4_)_2_SO_4_ and full squares: 1 M Na_2_SO_4_.

The last studied protease was trypsin (Figure 8). Figure 8a–c show the results using glyoxyl-trypsin. At pH 5.0, all salts additions on the buffer solution produced a stabilization of the enzyme, but this effect was not very significant. 1 M NaCl was slightly more stabilizing than 3 M NaCl, and 1 M Na_2_SO_4_ gave a slightly higher stabilization. This stabilization was lower using 1 M (NH_4_)_2_SO_4_ and increased using 3 M (NH_4_)_2_SO_4_ to levels close to 1 M Na_2_SO_4_. At pH 7.0, NaCl stabilized the enzyme, an effect which was more pronounced using 3 M than using 1 M. 1 M Na_2_SO_4_ stabilized the enzyme less than 1 M NaCl, while 1 M (NH_4_)_2_SO_4_ had a similar effect, which increased using 3 M (NH_4_)_2_SO_4_. At pH 9.0, 1 M NaCl was slightly destabilizing, while 3 M presented a stabilizing effect. 1 M Na_2_SO_4_ or (NH_4_)_2_SO_4_ were even more destabilizing than 1 M NaCl, and the enzyme inactivation in 3 M (NH_4_)_2_SO_4_ gave similar results to that in the presence of 1 M NaCl. We can conclude that glyoxyl-trypsin seemed not to be very responsible to the additions of salts in terms of stability. Figure 8d–f shows the results using glutaraldehyde-trypsin. At pH 5.0, NaCl was slightly positive for enzyme stability, more using 1 M than using 3 M. 1 M Na_2_SO_4_ was negative for enzyme stability, while 1 M and 3 M (NH_4_)_2_SO_4_ were significantly positive. At pH 7.0, NaCl was negative for enzyme stability at both concentrations, while Na_2_SO_4_ was positive (the only condition where stability was improved was using 1 M of this salt). The strongest effect, a destabilizing one, was found using (NH_4_)_2_SO_4_, and the effect was dramatic using 3 M of this salt, as the activity was fully destroyed after just 30 min. At pH 9.0, again everything was different: 1 M NaCl was positive for enzyme stability, while 3 M NaCl was negative. 1 M Na_2_SO_4_ and 1 M (NH_4_)_2_SO_4_ were also negative for enzyme stability in a similar way, while 3 M (NH_4_)_2_SO_4_ was again very negative for enzyme stability. That is, trypsin immobilized on glutaraldehyde was more sensible to the presence of high concentrations of salts than the glyoxyl immobilized enzyme.

That way, using proteases, the situation remains very dependent on the inactivation pH, the enzyme and the immobilization protocol. In this instance, the enzyme stability decreased in the presence of some salts more than in the case of lipases, and the stabilizing effects of 3 M (NH_4_)_2_SO_4_ is no longer a general rule, becoming in many instances a strong destabilizing agent. The increase of the salt concentration in some cases revert a negative effect in positive, while in other cases revert a positive effect in a negative one. The only rule that can be extracted from the results is that the effect of the salts on enzyme stability strongly depends on the specific case, suggesting that many different and opposite phenomena are simultaneous determining the final enzyme stability.

### 2.3. Effect of Different Salts on the Stability of Some Additional Immobilized Monomeric Enzymes

**Figure 9:**
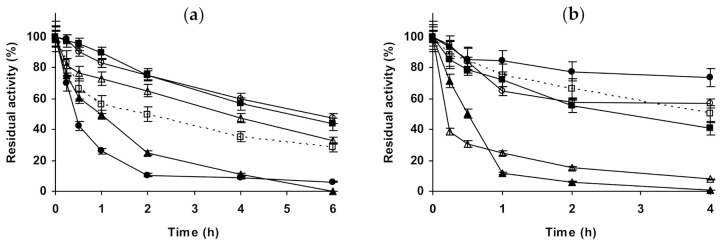
Effect of ionic strength and inactivation pH on the stability of glutaraldehyde-*β*-galactosidase. (**a**) pH 5.0 and 58 °C; (**b**) pH 7.0 and 58 °C. Other specifications are described in the Methods section. Dotted line, empty squares: references; full triangles: 1 M NaCl; empty triangles: 3 M NaCl; full circles: 1 M (NH_4_)_2_SO_4_; empty circles: 3 M (NH_4_)_2_SO_4_ and full squares: 1 M Na_2_SO_4_.

Here, using immobilized preparations, the effect of the salts on the stability of some additional monomeric enzymes have been analyzed. Starting with glutaraldehyde-*β*-galactosidase (Figure 9), at pH 5.0, 1 M NaCl produced a clear enzyme destabilization, while 3 M is clearly stabilizing. 1 M Na_2_SO_4_ stabilized the immobilized enzyme even more than 3 M NaCl, while the presence of 1 M (NH_4_)_2_SO_4_ was negative for enzyme stability and 3 M of this salt permitted to have a stability similar to that observed using as inactivating medium 1 M Na_2_SO_4_. At pH 7.0, the enzyme stability decreased when NaCl were added, the increase in its concentration accelerated the initial steps of the enzyme inactivation but the last activity fraction was more stable. Na_2_SO_4_ was not so negative for enzyme stability, as NaCl while 1 M (NH_4_)_2_SO_4_ permitted a higher enzyme stability and 3 M (NH_4_)_2_SO_4_ effect was slightly negative for enzyme stability. At pH 9.0, the study could not be performed due to the very poor stability of the enzyme even at room temperature.

**Figure 10:**
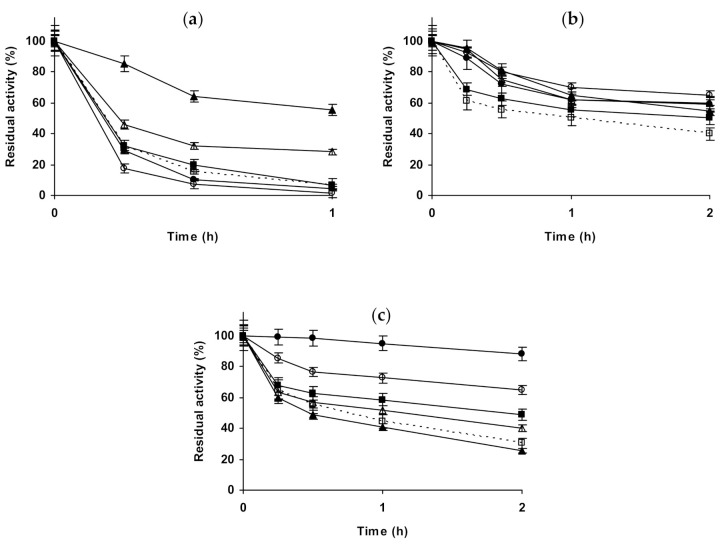
Effect of ionic strength and inactivation pH on the stability of glutaraldehyde-laccase. (**a**) pH 5.0 and 62 °C; (**b**) pH 7.0 and 59 °C; (**c**) pH 9.0 and 57 °C. Other specifications are described in the Methods section. Dotted line, empty squares: references; full triangles: 1 M NaCl; empty triangles: 3 M NaCl; full circles: 1 M (NH_4_)_2_SO_4_; empty circles: 3 M (NH_4_)_2_SO_4_ and full squares: 1 M Na_2_SO_4_.

The second studied immobilized monomeric enzyme was glutaraldehyde-laccase (Figure 10). At pH 5.0, 1 M NaCl significantly stabilized the enzyme, while using 3 M the enzyme stabilizing effect was less clear. 1 M Na_2_SO_4_ or (NH_4_)_2_SO_4_ did not present an effect on enzyme stability, while 3 M (NH_4_)_2_SO_4_ promoted a destabilization of the enzyme. At pH 7.0, 1 and 3 M NaCl produced similar stabilizing affects, while 1 M Na_2_SO_4_ was less stabilizing. 1 M (NH_4_)_2_SO_4_ stabilized the enzyme like NaCl, and using 3 M this effect was slightly improved. At pH 9.0, 1 M NaCl almost did not affect enzyme stability, while 3 M slightly improve it. The stabilizing effect was clearer using 1 M Na_2_SO_4_ and further increased using 1 M (NH_4_)_2_SO_4_, although this positive effect on enzyme stability decreased using 3 M of the salt. 

**Figure 11:**
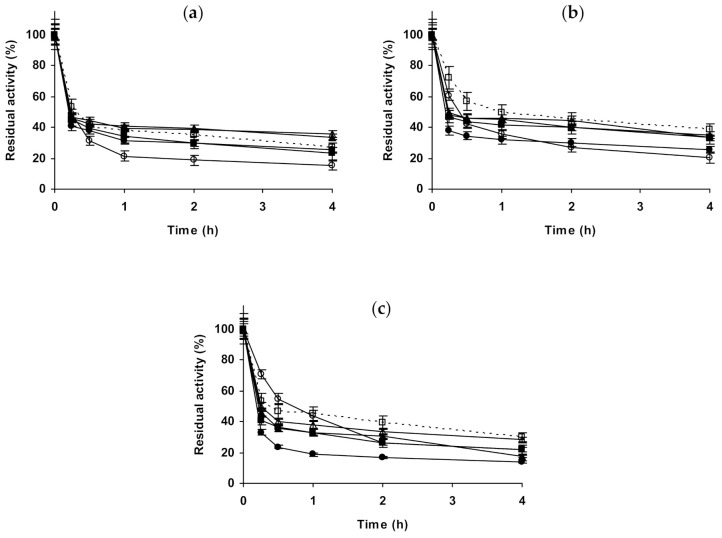
Effect of ionic strength and inactivation pH on the stability of glyoxyl-PGA. (**a**) pH 5.0 and 65 °C; (**b**) pH 7.0 and 65 °C; (**c**) pH 9.0 and 47 °C. Other specifications are described in the Methods section. Dotted line, empty squares: references; full triangles: 1 M NaCl; empty triangles: 3 M NaCl; full circles: 1 M (NH_4_)_2_SO_4_; empty circles: 3 M (NH_4_)_2_SO_4_ and full squares: 1 M Na_2_SO_4_.

The last studied enzyme in this section was glyoxyl-PGA (Figure 11), although it is a heterodimeric enzyme, it is really the result of an auto-processing of pre-enzyme, and it does not establish equilibrium between dissociated and associate subunits [112,113,114]. The effects of the salts in the stability of this enzyme are in general quite small. At pH 5.0, NaCl has a marginal stabilizing effect, similar at 1 and 3 M. 1 M Na_2_SO_4_ or (NH_4_)_2_SO_4_ presented not significant effect, while 3 M (NH_4_)_2_SO_4_ destabilized the enzyme. At pH 7.0, all salts have a negative effect on enzyme stability. NaCl was slightly negative at both, 1 and 3 M. 1 M Na_2_SO_4_ decreased the enzyme stability in a more significant way, and the stability decreased even more using 1 or 3 M (NH_4_)_2_SO_4_. Results at pH 9.0 were similar, except that using 3 M (NH_4_)_2_SO_4_ the first inactivation was slower than that in just buffer, but later gave less residual activity.

Thus, again a great diversity of results can be found for these enzymes, the pH determines in many instances if one specific salt has a positive or negative result.

### 2.4. Effect of Different Salts on the Stability of Some Glutaraldehyde-Amino Agarose Immobilized Multimeric Enzymes

**Figure 12:**
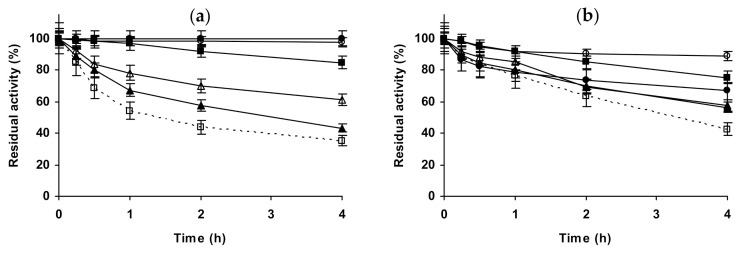
Effect of ionic strength and inactivation pH on the stability of glutaraldehyde-*β*-glucosidase. (**a**) pH 5.0 and 54 °C; (**b**) pH 7.0 and 62 °C. Other specifications are described in the Methods section. Dotted line, empty squares: references; full triangles: 1 M NaCl; empty triangles: 3 M NaCl; full circles: 1 M (NH_4_)_2_SO_4_; empty circles: 3 M (NH_4_)_2_SO_4_ and full squares: 1 M Na_2_SO_4_.

Figure 12 shows the effects of the different salts in the stability of the immobilized dimeric *β*-glucosidase preparation. At pH 5.0, NaCl produced a stabilizing effect that increased when increased the salt concentration. 1 M Na_2_SO_4_ produced a more significant stabilizing effect, while in the presence of 1 M and 3 M (NH_4_)_2_SO_4_ the enzyme retained the full initial activity during the whole inactivation time. At pH 7.0, NaCl has a shorter stabilizing effect than at pH 5.0, and similar using both concentrations. 1 M Na_2_SO_4_ produced a higher stabilization than NaCl, while 1 M (NH_4_)_2_SO_4_ gave a value similar to NaCl, however, the maximum stability of this enzyme preparation was found using 3 M (NH_4_)_2_SO_4_. At pH 9.0, the stability of the immobilized enzyme was too low to give reliable data.

**Figure 13:**
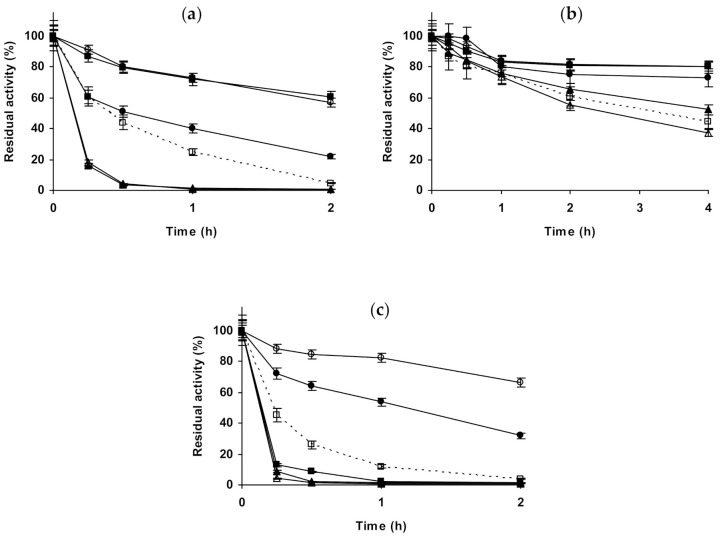
Effect of ionic strength and inactivation pH on the stability of glutaraldehyde-catalase. (**a**) pH 5.0 and 50 °C; (**b**) pH 7.0 and 50 °C; (**c**) pH 9.0 and 47 °C. Other specifications are described in the Methods section. Dotted line, empty squares: references; full triangles: 1 M NaCl; empty triangles: 3 M NaCl; full circles: 1 M (NH_4_)_2_SO_4_; empty circles: 3 M (NH_4_)_2_SO_4_ and full squares: 1 M Na_2_SO_4_.

The last studied enzyme was an immobilized tetrameric catalase (Figure 13). At pH 5.0, NaCl behaved as a strong destabilizing agent, at both concentrations. However, 1 M or 3 M Na_2_SO_4_ produced a similar and significant stabilization, while 1 M (NH_4_)_2_SO_4_ has a short stabilizing effect. At pH 7.0, the effect of NaCl on enzyme stability was low, slightly positive at 1 M and slightly negative at 3 M, while 1 M Na_2_SO_4_ remained as a stabilizing condition, 1 M (NH_4_)_2_SO_4_ produced a small stabilization, and 3 M promoted an enzyme stability similar to 1 M Na_2_SO_4_. The situation changed again at pH 9.0, NaCl becoming a very destabilizing agent for this enzyme, similar to Na_2_SO_4_ (in opposition to the results found at pHs 5.0 and 7.0). However, (NH_4_)_2_SO_4_ promoted the enzyme stabilization, and this effect was higher when the salt concentrations increased. That is, the effect of the salts strongly changed when the inactivation pH changed.

Again, the effects of the salts follow no clear rules, and the same salt may be positive in one condition and negative in other.

## 3. Materials and Methods

### 3.1. Materials 

CALA (NovoCor^®^ AD L, 7.63 mg of protein/mL), CALB (Lipozyme^®^ CALB L, 5.57 mg of protein/mL), EVT (Eversa^®^ Transform 2.0, 27.5 mg of protein/mL), RML (Palatase^®^ 2000 L, 2.67 mg of protein/mL) and laccase (Novozym^®^ 51033, 39.86 mg of protein/mL) were kindly donated by Novozymes (Madrid, Spain). Ficin (79 mg of protein/mL) was produced as previously described [115]. *β*-galactosidase (20 units of *o*NPG/mg of protein), *β*-glucosidase (350 mg of protein/g of powder), catalase (470 mg of protein/g of powder), CRL (32 mg of protein/g of powder), chymotrypsin and trypsin (lyophilized powder), PGA (68 mg of protein/mL), 6-nitro-3-(phenylacetamido)benzoic acid (NIPAB), Nα-benzoyl-D,L-arginine *p*-nitroanilide hydrochloride (BAPNA), N-benzoyl-L-tyrosine *p-*nitroanilide (BTPNA), *o*-nitrophenyl *β*-D-galactopyranoside (*o*NPG), *p*-nitrophenyl, *β*-D-glucopyranoside (*p*NPG), *p*-nitrophenyl butyrate (*p*NPB) and sodium borohydride were purchased from Sigma Aldrich (Alcobendas, Spain). 2,2′-azino-bis (3-ethylbenzothiazoline-6-sulfonic acid) diammonium salt (ABTS^®^) was acquired from Roche (Mannheim, Germany). Horseradish peroxidase (2680 U/mg of powder) was purchased from Orion High Technologies (Parla, Spain). Bradford’s method was used for the protein concentration determination, using bovine serum albumin as standard [116]. Octyl-Sepharose^®^ Cl-4B beads were purchased from GE Healthcare (Madrid, Spain) and 4% BCL Agarose Beads Standard were purchased from ABT (Alcobendas, Spain). Agarose beads were used to produce aminated agarose [117,118], and then modified with glutaraldehyde to produce the glutaraldehyde-amino agarose activated support [119,120,121]. All other reagents were of analytical grade. 

### 3.2. Methods

The experiments were performed in triplicate and the data are given as mean values and standard deviation. 

#### 3.2.1. Enzyme Activity Assay

One Unit (U) of activity was defined as the amount of enzyme that hydrolyzes 1 μmol of substrate per minute under the specified conditions. Enzyme activity was determined using a spectrophotometer with magnetic stirring at 200 rpm, at a temperature of 25 °C (except for ficin, where the assay was performed at 55 °C).

Lipases activity was quantified by determining the variance in absorbance at 348 nm produced by the release of *p*-nitrophenol (isosbestic point, *ε* under these conditions is 5150 M^−1^cm^−1^ [122]) in the hydrolysis of 50 µL of 50 mM *p*NPB in 25 mM sodium phosphate at pH 7.0. The reaction was started by adding 50 µL of lipase solution or suspension to 2.5 mL of buffer containing *p*NPB.

Ficin and trypsin activities were determined by measuring the change in absorbance at 405 nm caused by the release of *p*-nitroaniline (*ε* under these conditions is 9960 M^−1^cm^−1^ [123]), produced by the hydrolysis of BAPNA. For ficin substrate, it was prepared at 1 mM in 100 mM phosphate at pH 7.0, containing 5 mM cysteine and 5 mM EDTA and 200 μL of enzyme solution or suspension were added to 2.5 mL at incubated at 55 °C for 15 min [115]. When it was used as trypsin, BAPNA was prepared at a concentration of 2 mM in 50 mM sodium phosphate at pH 7.0 containing 30% ethanol [124] adding 50–200 μL of enzyme solution or suspension to 2.5 mL of BAPNA solution to start the reaction. 

Chymotrypsin activity was determined by measuring the variation in absorbance at 386 nm produced by the release of *p*-nitroaniline (*ε* under these conditions is 12,500 M^−1^ cm^−1^ [125]), produced by the hydrolysis of BTPNA. A 40 mM BTPNA stock solution was prepared in DMSO. The reaction was started by adding 200 μL of enzyme solution or suspension to 75 μL of 40 mM BTPNA solution in 2.5 mL of 100 mM sodium phosphate at pH 7.0 containing 40% ethanol. 

*β*-Galactosidase activity was measured by the change in absorbance at 380 nm produced by the release of *o*-nitrophenol (*ε* under these conditions is 10,493 M^−1^cm^−1^ [126]) in the hydrolysis of 10 mM *o*NPG in 100 mM sodium acetate at pH 4.5. 50–100 μL of enzyme solution or suspension were added to 2.5 mL of the substrate solution to initialize the reaction.

The laccase activity was determined by recording the increase in absorbance at 420 nm produced by the oxidation of the ABTS^©^ (*ε* under these conditions is 36,000 M^−1^cm^−1^ [76]). The reaction was started by adding 25 μL of enzyme solution or suspension to a solution composed by 1 mL of 100 mM sodium acetate at pH 5.0 and 1 mL of 50 mM ABTS^©^ in water. 

Activity of PGA was measured using NIPAB as substrate as described by Kutzbach et al. [108]. The assay was performed continuously following the increase of absorbance at 405 nm (*ε* under these conditions is 8730 M^−1^cm^−1^ [108]). The reaction was started by adding 100 μL of enzyme solution or suspension to 2 mL of 0.15 mM NIPAB in 50 mM sodium phosphate at pH 7.5 solution.

*β*-Glucosidase activity was measured by the increase in absorbance produced at 380 nm by the release of *p*-nitrophenol (*ε* under these conditions is 3459.3 M^−1^cm^−1^ [120]) caused in the hydrolysis of *p*NPG. The reaction was started by adding 120 μL of enzyme solution or suspension to a solution composed by 200 μL of 10 mM *p*NPG prepared in 100 mM sodium phosphate at pH 7.0 and 1.68 mL of 100 mM sodium phosphate at pH 7.0. 

The catalase activity was determined by measuring the reduction in the absorbance at 240 nm promoted by the consumption of hydrogen peroxide concentration in the reaction medium (the calculated *ε* under these conditions is 32.7 M^−1^cm^−1^). It consisted of 200 μL of 200 mM H_2_O_2_ added to 2.25 mL of 100 mM sodium phosphate, pH 7.0, 50 μL of enzyme solution or suspension were added to start the reaction. 

#### 3.2.2. Enzymes Immobilizations

All immobilizations were performed following the activity of supernatant, suspension and a reference of the enzyme under identical conditions, immobilization yield was in all cases very next to 100% [127].

##### Immobilization of the Lipases on Octyl Agarose Beads

Lipases immobilizations were performed using 1 (CALA, CALB, CRL, RML) or 0.2 (EVT) mg of enzyme/g of wet support to prevent diffusion limitations and protein-protein interactions [128]. The stock enzyme solution was prepared in 5 mM sodium phosphate at pH 7.0 and 25 °C. The support was added in a proportion of 1 g/10 mL of the enzyme solution. The activity was measured using *p*NPB to determine immobilization yield and expressed activity. After immobilization, the biocatalysts were washed with water, vacuum dried to eliminate inter-particle water and stored at 6–8 °C. 

##### Immobilization of Enzymes on Glutaraldehyde-Amino Agarose Beads

The agarose beads were modified with ethylenediamine to produce aminated agarose as previously described [117,118]. The glyoxyl agarose support was activated using 10% glutaraldehyde prepared in 200 mM sodium phosphate solution at pH 7.0 and left overnight as previously described [119]. Immobilizations on glutaraldehyde-amino agarose were performed using 1 g of support per 10 mL of enzyme solution prepared 5 mM sodium phosphate at pH 7.0 at 25 °C. The immobilization courses were followed during 24 h. Finally, the immobilized enzymes were washed, vacuum dried and stored at 6–8 °C. The enzyme loadings of the immobilized biocatalysts were: CALA and CALB at 2 mg of enzyme/g of support [111], CRL at 1 mg of enzyme/g of support, RML at 4 mg of enzyme/g of support and EVT at 0.4 mg of enzyme/g of support, ficin at 10 mg of enzyme/g of support [129], chymotrypsin at 15 mg of enzyme/g of support and trypsin at 3 mg of enzyme/g of support (3 mM of benzamidine was added to prevent autolysis [130]), *β*-galactosidase at 1 mg of enzyme/g of support [121], laccase at 20 mg of enzyme/g of support [76], *β*-glucosidase at 49 mg of enzyme/g of support [120], catalase at 0.5 mg of enzyme/g of support [131].

##### Immobilization of the Enzyme on Glyoxyl Agarose Beads

Ficin was immobilized using 10 mg of enzyme/g of support, while chymotrypsin and trypsin (in the presence of 3 mM benzamidine [130]) were immobilized using a loading of 3 mg enzyme/g of support, in 100 mM sodium bicarbonate at pH 10.05 and 25 °C, using the protocols previously described [100,115,132,133]. PGA was immobilized using a support load of 5 mg enzyme/g of support. The enzyme was added to a solution composed of 100 mM sodium carbonate containing 100 mM phenyl acetic acid and 20% glycerol to prevent enzyme inactivation at pH 10.05 [134,135]. After enzymes immobilizations, 1 mg/mL of solid sodium borohydride was added and the immobilization suspensions were stirred for 30 min. After, the biocatalysts were washed with water, vacuum dried to eliminate inter-particle water and stored at 6–8 °C.

#### 3.2.3. Stress Inactivation of Different Enzyme Preparations in the Presence of Different Salts on Biocatalyst Stability

The effect of different concentrations of different salts on the stability of all immobilized biocatalysts was studied under different conditions of salts concentration and pHs. Each enzyme was incubated in 100 mM sodium acetate at pH 5.0, 100 mM Tris HCl at pH 7.0 (phosphate was no used by its negative effects on immobilized lipases and galactosidase stabilities [73,76]) or 100 mM sodium carbonate at pH 9.0 and in some instances 1 M or 3 M of NaCl; or 1 M or 3 M of (NH_4_)_2_SO_4_; 1 M of Na_2_SO_4_, was added. Inactivation temperatures for each enzyme were selected for each enzyme and inactivation pH value to get inactivation rates of the enzyme (when inactivated in just buffer) that yielded reliable inactivation courses. This way, an easy comparison of the effect of the different salt additions on its stability may be performed.

## 4. Conclusions

As stated in the Introduction, there are many reports in the literature discussing the likely role of high concentrations of salts on enzyme stability, in an attempt to achieve a mechanistic explanation to these effects. Using free enzymes, the experimental studies are very few because of the possibility of enzyme aggregation, which can make the understanding of the phenomena difficult. That way, most previous studies are mainly theoretical ones. These theoretical papers, involving one or two enzymes, reached different conclusions, some stating that the ionic strength should be negative for enzyme stability, some others stating that it should be positive. In these papers, the conclusions are “clear”, but opposite depending on the enzyme or conditions. These theoretic studies usually conclude that the effect of the high concentrations of salts on enzyme stability should be negative, while we have experimentally found that some salts may greatly improve enzyme stability, although other salts in other conditions may be strong destabilizing agents.

Thanks to the use of a wide variety of enzymes, inactivation conditions and immobilization protocols, the conclusions reached in this new paper on the effects of ion strength on enzyme stability may be considered quite strong. The effect of high ion strength on immobilized enzyme stability may be positive or negative, depending on the enzyme, immobilization protocol and inactivation conditions. That way, the results discussed in this paper show, using 13 different immobilized enzymes, including monomeric and multimeric enzymes, in many instances immobilized following two different protocols, that we are very far from understanding all the phenomena occurring on enzyme inactivation and how that presence of high concentrations of salts may alter this. That is, trying to reduce the effects of the salts on enzyme stability to simple mechanistic phenomenon may be incorrect in the current situation of the enzyme inactivation mechanisms. These simplified models can explain what occurs with a single enzyme, at a single inactivation pH and immobilized following a specific protocol. However, with the available technologies, the researcher cannot, nowadays, be in a condition to predict the effects of a specific salt in a specific immobilized enzyme under some given inactivation condition, which means that this must be empirically studied. 

In fact, the results presented in this paper confirm the ones that could be understood from the general reading of previous papers on this matter, the effect of a high concentration of salt on the stability of a specific enzyme may be positive or negative, depending on many factors, which very likely are interacting among them. This effect strongly depends on the properties of each specific enzyme (the ionic bridges that can stabilize active or partially inactivate structures, possible relevance of some specific ions for enzyme stability, the ordering of the water molecules that can reduce enzyme mobility, the solubility of the enzyme groups that go from internal pockets to the outside, etc.). 

This is more complex considering that the effect of a specific salt will depend on its concentration; it may be negative at one concentration and positive if using a higher concentration or vice versa. This shows that there are several effects acting in a simultaneous way determining the enzyme stability in these media. The ordering of the water molecules should decrease the freedom of movement of the enzyme moieties, increasing thus enzyme stability. However, in many instances we have detected a negative effect of the presence of salts on enzyme stability that increases with salt concentration, suggesting that other facts are also relevant (see introduction). The effects also strongly depend on the salt nature. However, this effect is not obvious, as sulfate salts may be positive or negative for a specific enzyme, while NaCl could be negative for enzyme stability when sodium sulfate was the most positive studied salt. In this sense, both intensity of the effect on enzyme stability and sense of this effect, depend on the inactivation pH.

A new factor, not previously reported, is how the enzyme immobilization protocol greatly affects the effects of the salts on enzyme stability. However, once again, there is not a clear rule stating that one immobilization protocol will give final biocatalysts more responsive to the presence of these high concentrations of salts. Although with lipases (NH_4_)_2_SO_4_ seems to be the agent that usually produces the highest stabilization of the immobilized enzymes under all conditions, this is not confirmed using other enzymes. In many instances NaCl or Na_2_SO_4_ permitted to achieve the highest enzyme stabilities.

Regarding the relation of the inactivation pH with the presence of salt on the effects on enzyme stability, usually (again not a universal rule) the stabilizing effects may be more frequently found at pH 7.0 or 5.0, while at pH 9.0 it is very frequent to observe a very negative effect of the salts. The exact reason for each result would require deep modelling analysis, and even this may be complex considering the effect of the immobilization protocol, that will produce enzymes with different and unknown structures. 

However, the current paper shows that it is possible to ensure that the presence of the salts used in this study, in absence of possible enzyme aggregations, has a effect on enzyme stability, very high in some instances, while in other cases it may be weaker, but always significant. This effect may be stabilizing or destabilizing. The exact correlation of this effect with one specific feature of the salt or of the enzyme seems very risky, as a strong correlation of many parameters (and some of them may be fully unknown to date) seem to be relevant for the final result. That way, only experimentally studying the effects of the salts on enzyme stability, the researcher will be sure of the real nature of these effects (but not on the exact causes for these effects).

## Data Availability

All data are reported in the paper, any specific query may be addressed to rfl@icp.csic.es.

## Sample Availability

Samples of the compounds are available from the authors.
